# Scientific Abstracts 7th Congress of the European College of Equine Internal Medicine

**DOI:** 10.1111/jvim.12585

**Published:** 2015-05-21

November 7–8, 2014

Prague, Czech Republic

**Table:** 

**Scientific Programme**		
**Friday 7^th^ of November**		
8:00 – 9:00	Kevin Corley	Critical care at the edge ‐ how I quack and what is the evidence for my quackery?
9:00 – 9:15	S.E. Ross	Are equine practitioners in the UK using antimicrobials responsibly? (Liphook, UK)
9:15 – 9:30	E. de Graaf‐Roelfsema	Familial spinal ataxia in three haflinger foals (Utrecht, NL)
9:30 – 9:45	N.M. Collins	Admission severe hyponatraemia in hospitalised foals (Scone, AUS)
9:45 – 10:00	D.A. Kingston	Trypanosomosis in working equids in west Africa (Glasgow, UK)
10:30 – 11:30	Monica Aleman	Neurologic, neuromuscular and muscle disease in foals
11:30 – 12:30	Ales Tomek	Neurologic, neuromuscular and muscle disease in puppies
13:30 – 14:30	Thomas J. Divers	Urinary disorders in foals
14:30 – 15:30	Ben Sykes	EGUS ECEIM Consensus
16:00 – 17:30		ECEIM General Assembly
17:30 – 18:30		ECEIM Resident Meeting
**Saturday 8th of November**		
9:00 – 10:00	Dennis E. Brooks	Ocular manifestation of systemic disease
10:00 – 10:15	F. Bonelli	Procalcitonin in healthy and endotoxemic horses (Pisa, ITA)
10:15 – 10:30	C. Cesarini	Association between antemortem hemostatic profile and postmortem fibrin deposits in horses with colic (Barcelona, ESP)
10:30 – 10:45	M. Duz	NSAIDs, diarrhoea and big data (Glasgow, UK)
10:45 – 11:00	O. Kutasi	Evaluation of lipase and amylase levels in the diagnostic investigation for the equine acute abdomen (Budapest, HU)
11:30 – 12:30	Richard Piercy	Pathophysiology of recurrent laryngeal neuropathy
12:30 – 12:45	F. Malalana	Assessment of cervical oesophageal motility by transcutaneous ultrasonography in horses (Liverpool, UK)
12:45 – 13:00	M. Wehrli Eser	Short and long‐term survival rate of horses after large intestinal colic surgery – Preliminary results (Zurich, CH)
13:00 – 13:15	R.C. Jago	Comparison of body weight loss in surviving and non‐surviving cases of chronic equine grass sickness (Edinburgh, UK)
13:15 – 13:30	A. Schoster	The effect of a newly designed probiotic on prevention of equine neonatal diarrhea (Zurich, CH)
14:30 – 14:45	Z. Drabkova	Retrospective study of twenty equine lymphomas (Brno, CZE)
14:45 – 15:00	D. De Clerq	Effect of commonly used electrode configurations on electrocardiographic parameters in the standing horse: Preliminary results (Ghent, BE)
15:00 – 15:15	B. Broux	Sotalol hydrochloride as an oral anti‐arrythmic in the horse? (Ghent, BE)
15:15 – 15:30	M.J.P. Theelen	Hemochromatosis and liver failure in 11 horses due to chronic iron intoxication (Utrecht, NL)
15:30 – 16:30	Lutz Goehring	Infectious encephalopathies in Europe
17:00 – 17:15	A. Bonetto	Tetanus immunity in horses in northern Germany (Hannover, GER)
17:15 – 17:30	M. Robin	The effect of the topical application of deltamethrin to horses on blood‐feeding by *Culicoides* midges (Liverpool, UK)
17:30 – 17:45	T.M. Forhdham	Fibrosis in white adipose tissue of horses with EMS and PPID (Glasgow, UK)
17:45 – 18:00	R.A. Morgan	Predicting laminitis risk in horses with endocrine disease (Glasgow, UK)
18:00 – 19:00	Thomas J. Divers	Hepatoencephalopathy

**Table:** 

**Poster Session**	
1.	Ultrasound‐Guided Percutaneous Transcatheter Delivery of An Occlusion Device in Two Horses
	G. van Loon
2.	Successfull Treatment of Dermatographism With Cetirizine in A Horse
	A.J. Van den Brom‐Spierenburg
3.	Relationship between Body Dimension, Body Weight, Age, Gender, Breed and Echocardiographic Dimensions in Young Endurance Horses
	D.S. Trachsel
4.	Pharmacokinetics of Five Commercially Available Formulations of Omeprazole
	B.W. Sykes
5.	Influences of Age and Sex on Leukocytes of Healthy Horses and Their *Ex Vivo* Cytokine Release
	C. L. Schnabel
6.	Neuromodulation Using Percutaneous Electrical Nerve Stimulation; An Effective and Safe Therapy For The Management of Trigeminal‐Mediated Headshaking in Five Horses
	V.L.H. Roberts
7.	A Prospective Clinical Evaluation of The Comparative Efficacy of Three Trypanocides in The Treatment of Equine Trypanosomiasis in The Gambia
	A.G. Raftery
8.	Idiopathic Peritonitis in Horses: Clinical Findings and Long Term Survival in 58 Cases
	J. Pringle
9.	Microbiological and histological Analysis of Equine Skin Cores. Impact of Cannula Size and Injection Site Preparation, For Intramuscular Injection Complications
	T. Puschmann
10.	In‐Vivo Measurement of Muscle Protein Synthesis in The Horse
	R.J. Naylor
11.	Detection of Equine Herpes Virus 5 in Different Respiratory Samples of Horses
	L. Moravszki
12.	Clinical Findings, Treatment and Resolution of Fluroxypyr Toxicity in Three Horses
	J.I. Michutta
13.	Glucagon Curve and Insulin‐Glucagon Molar Ratio in Healthy Adults Donkeys Under Intravenous Glucose Challenge
	F.J. Mendoza
14.	Grazing Horses are Exposed to Terrestrial Cyanobacteria
	B. C. McGorum
15.	HDL, LDL and Total Cholesterol in Healthy and Septic Neonatal Foals
	J. Mariella
16.	Kaolin Activated Thromboelastography in 20 Healthy Horses
	K. Machackova
17.	Ultrastructural Mitochondrial Alterations in Equine Myopathies of Unknown Origin
	K. Van Driessche
18.	The Effect of Feeding on the Repeatability of Right Dorsal Colonic Wall Thickness Measurement
	N. Kerbyson
19.	Exercise Indused Pulmonary Hamorrhage (EIPH) in Trotters: Impact on Later Performance
	C.F. Ihler
20.	Effects of Different Catecholamines on Myocardial Function in Isoflurane Anaesthetized Horses
	C. Hopster‐Iversen
21.	Equine Procalcitonin As A Potential Inflammatory Biomarker in Horses
	Teschner D.

## 0001

### ARE EQUINE PRACTITIONERS IN THE UK USING ANTIMICROBIALS RESPONSIBLY?

**S.E. Ross, D.I. Rendle.**

Liphook Equine Hospital, Forest Mere, Liphook, Hampshire, GU30 7JG, United Kingdom

**Reasons for performing study:** In order to inform veterinary surgeons of the importance of responsible antimicrobial use, an understanding of current habits is required. Previous questionnaire studies have not established actual behaviour in clinical practice.

**Purpose:** (i) Determine the frequency with which protected antimicrobials are being used when first‐line choices would suffice. (ii) Determine whether appropriate doses of systemic antimicrobials are being administered. (iii) Investigate clinician factors that may influence prescribing habits.

**Methods:** Patient records for 113 horses referred to Liphook Equine Hospital for the treatment of limb wounds between January 2012 and October 2013 were reviewed. Referring veterinary practices were contacted for further clinical information.

**Results:** Systemic antimicrobials were administered prior to referral in 94/113 (83.2%) horses. Of these, 83/94 (88.3%) received “first‐line” antimicrobials (as defined by The British Equine Veterinary Association*). However, 8/94 (8.5%) received “protected” antimicrobials; 4 horses were treated with 3rd or 4th generation cephalosporins and 4 horses were treated with fluoroquinolones. Fifty‐one of 90 (56.6%) horses received a sub‐optimal dose of at least one antimicrobial. More experienced practitioners were less likely to administer protected antimicrobials. There was no relationship between post‐graduate training or type of practice and the use of protected antimicrobials or the administration of sub‐optimal doses.

**Conclusions and practical applications:** The results of this study highlight the need for greater awareness of, and compliance with, recommendations for the use of antimicrobials in equine practice. Under‐dosing is a risk factor for the development of antimicrobial resistance and appears to be commonplace.

*http://beva.org.uk/_uploads/documents/beva-antimicrobial-policy-template-2012.1.pdf

## 0002

### FAMILIAL SPINAL ATAXIA IN THREE HAFLINGER FOALS: A GENETIC CONDITION?

**E.de Graaf‐Roelfsema^1^, W. Bergmann^2^, I.D. Wijnberg^1^, C. Drögemüller^3^**

^1^Department of Equine Sciences, Faculty of Veterinary Medicine, Utrecht University, Utrecht, the Netherlands, ^2^Department of Pathobiology, Faculty of Veterinary Medicine, Utrecht University, Utrecht, the Netherlands, ^3^Institute of Genetics, Vetsuisse Faculty, University of Bern, Bern, Switzerland

A 6 week old Haflinger filly showed progressive spinal ataxia in all four limbs with generalized neuropathic changes on EMG. X‐rays of spinal column were unremarkable. Plasma vitamin E levels were decreased in mare and filly. Two other foals were born with same symptoms and time of onset (filly from same parents and one colt from same father, different mother). Due to the progressive nature of the disease the filly was euthanized at 1.5 years of age. On necropsy, gross lesions were limited to a caecal cyathostominae infection.

Histopathology showed bilateral, symmetric demyelination of the dorsal spinocerebellar tracts of the thoracic spinal cord. All spinal funiculi presented with mild Wallerian degeneration. There was moderate to severe gliosis of the grey matter of spinal cord and brain. Few spinal neurons contained lipofuscin. These pathomorphologic changes were compatible with a chronic course of neuroaxonal dystrophy.

The pedigrees of the three affected foals suggested a monogenic autosomal recessive inheritance and all affected animals trace back to a single male ancestor. Therefore, it is highly likely that the condition is inherited. Genotyping of 62k SNPs in two cases and seven healthy relatives including the parents revealed several potentially associated genome regions. Furthermore, whole genome sequencing of a single case was performed revealing 134 DNA variants with a significant effect on the encoded proteins which are present in homozygous state in the affected animal only.

## 0003

### RETROSPECTIVE STUDY OF TWENTY EQUINE LYMPHOMAS

**R. Bodnár^1^, Z. Drábkova^1^, B.C. Schwarz^2^, O. Kutasi^3^, B. Bezděková^1^**

^1^Equine Clinic, Faculty of Veterinary Medicine, University of Veterinary and Pharmaceutical Sciences Brno, Czech Republic, ^2^Pferdeklinik Altforweiler, Germany, ^3^Equine Clinic, Faculty of Veterinary Science, Szent Istvan University, Hungary

The purpose of this retrospective multicenter clinical study was to make a general survey of the equine lymphoma population.

Twenty cases of equine lymphoma were found between 2003 and 2014. The prevalence of the studied population was 0.08%. Multicentric lymphoma represented the major type 15/20 (75%) followed by alimentary 3/20 (15%) and mediastinal 2/20 (10%). The B‐form 5/8 (63%) prevailed over the T‐form 3/8 (37%) and 3/5 (60%) of the multicentric B‐form were TCRLBCL. 15/20 (75%) of the cases were males and 10/20 (50%) of the cases were under 5 years of age but no risk factors have been statistically confirmed. The main presenting complaints were anorexia 11/20 (55%) and weight loss 9/20 (45%). Cachexia 12/20 (60%), tachycardia 10/20 (50%) and fever 8/20 (40%) were the most common clinical signs. The most frequent laboratory findings were leukocytosis 12/18 (67%), hypoalbuminaemia 12/18 (67%) and hyperproteinaemia 11/19 (58%). Leukemia was found in only two cases 2/20 (10%).The most common abnormality found on ultrasonography was peritoneal 10/15 (67%) and pleural 8/13 (62%) free fluid. Cytology of the pleural fluid revealed the presence of neoplastic cells in 6/6 (100%) and of the peritoneal fluid in 3/4 (75%) cases. Mass was found during rectal examination in 7/14 (50%) cases and correlated in 7/7 (100%) with the postmortem findings of neoplasia. Lymphoma was invariably fatal.

Thoracic and abdominal ultrasonography, cytology of the effusion and rectal examination proved to be the most effective diagnostic tests for equine lymphoma in our study.

## 0004

### TRYPANOSOMOSIS IN WORKING EQUIDS IN WEST AFRICA: CHARACTERISING NEUROLOGICAL DISEASE

**D.A. Kingston^1^, J. Rodgers^1^, S. Sharpe^1^, P. Capewell^1^, W. Weir^1^, L. Morrison^2^, P. Kennedy^3^, B. Bradley^1^, D.G.M. Sutton^1^**

^1^Institute of Biodiversity, Animal Health and Comparative Medicine, School of Veterinary Medicine, College of Medical, Veterinary and Life Sciences, University of Glasgow, United Kingdom, ^2^The Roslin Institute, Royal Dick School of Veterinary Studies, University of Edinburgh, Division of Infection and Immunity, Easter Bush, Midlothian, United Kingdom, ^3^Institute of Infection, Immunity & Inflammation, College of Medical, Veterinary and Life Sciences, University of Glasgow, United Kingdom

Equine CNS trypanosomosis is a multifocal neurological syndrome of equids which is often fatal. The condition is of international importance with widespread distribution and represents a neglected disease of welfare concern requiring further characterisation.

In this study, the clinical, pathological and molecular features of CNS trypanosomosis were investigated using cases presenting in The Gambia. Characterisation of the genotypic variation in the causative *T. brucei* subspecies (*T.b. evansi*, or *T.b. brucei*) was a novel specific aim. Full clinical and neurological examination was performed in working equids presenting with the disease, with detailed pathological and molecular assessment in 6 cases that required euthanasia.

Brain and spinal cord specimens (cranial cervical, cervical intumescence, caudal thoracic and lumbosacral) were preserved for evaluation. The severity of specific histopathological changes was scored: perivascular cuffing in the white and grey matter, meningitis, oedema and cellularity in the white and grey matter. DNA extraction and subsequent PCR was performed on CNS samples with standard *T.brucei* primers and a microsatellite marker panel.

*T. brucei* DNA was present within the CNS of all clinical cases. Histopathology showed diffuse lymphocytic‐plasmacytic meningoencephalomyelitis with marked perivascular cuffing. Pathology was most severe in the white matter. Immunohistochemistry confirmed *T.brucei* spp within the CNS tissue sections. Initial microsatellite results indicate at least 4 *T. brucei* spp. genotypes in the study population, with the same blood and brain populations.

Parasitic genotypic variation will be used to assess most likely mode of parasite multiplication, and transmission. This will facilitate the design of international disease control measures for this devastating condition.

## 0005

### PROCALCITONIN IN HEALTHY AND ENDOTOXEMIC HORSES

**F. Bonelli^1^, V. Meucci^2^, T.J. Divers^2^, E. Jose‐Cunilleras^3^, M. Corazza^1^, R. Tognetti^1^, G. Guidi^1^, L. Intorre^1^, M. Sgorbini^1^**

^1^Department of Veterinary Sciences, via Livornese snc, 56122, San Piero a Grado PI, Italy, ^2^College of Veterinary Medicine, Cornell University, Vet Box 25, Ithaca, NY 14853, USA, ^3^Department of Animal Medicine and Surgery, Universitat Autonoma de Barcelona, Bellaterra, Spain

Procalcitonin (PCT) seems to be an early marker of bacterial infection. The increase of its concentration is due to bacterial endotoxin and inflammatory cytokines. In horses’ GI diseases, hypersecretion of fluid, motility disturbances, altered microbial flora and impaired mucosal barrier may lead to absorption of endotoxin and/or bacterial products through the compromised mucosa. The aim of this work was to evaluate the plasmatic PCT concentration in healthy horses and those with acute GI diseases, in order to evaluate the differences between the two groups.

Plasma PCT concentration was evaluated in 45 horses referred to three different University Teaching Hospitals, and in 16 healthy horses that underwent similar management conditions. The following data were recorded in order to divide horses in healthy (*N* = 16), less than two criteria met, and clinical endotoxemic horses’ group (*N* = 45), two or more criteria met: neutropenia and/or toxic changes, increased PCV and TP, tachycardia, tachypnea, abnormal mucous membrane status and capillary refill time. Plasma PCT concentrations were measured with an equine PCT ELISA assay. Results were expressed as mean and standard deviation. T‐test for unpaired data was performed between healthy and suspected endotoxemic horses’ group. PCT concentration in healthy and clinical endotoxemic horses’ group was 22.3 ± 21.4 and 182.5 ± 83.9 pg/mL, respectively. T‐test showed differences between the two groups (*P* < 0.0001).

Our results showed an increase in plasma PCT concentration in clinical endotoxemic horses as reported in human medicine. PCT could be used in the equine practice for early therapy planning, in order to improve prognosis and restrain therapy costs.

## 0006

### ASSOCIATION BETWEEN ANTEMORTEM HEMOSTATIC PROFILE AND POSTMORTEM FIBRIN DEPOSITS IN HORSES WITH COLIC

**C. Cesarini^1^, M. Cotovio^2^, J.Ríos^3^, L. Armengou^1^, E. Jose‐Cunilleras^1^**

^1^Servei de Medicina Interna Equina, Fundació Hospital Clínic Veterinari and Departament de Medicina i Cirurgia Animals, Facultat de Veterinària, Universitat Autònoma de Barcelona, Barcelona, Spain, ^2^CECAV, Centro de Ciência Animal e Veterinária and Departamento de Ciências Veterinárias, Universidade de Trás‐os‐Montes e Alto Douro, Quinta de Prados, 5000‐801 Vila Real, Portugal, ^3^Laboratory of Biostatistics & Epidemiology, Universitat Autònoma de Barcelona; Statistics and Methodology Support Unit, IDIBAPS, Hospital Clínic, Barcelona, Spain

Disseminated intravascular coagulation (DIC) is frequent in horses with severe gastrointestinal disorders. Antemortem diagnosis is based on clinicopathological hemostatic results and clinical signs. Postmortem studies have found fibrin microthrombi in tissues of these horses, but studies relating these findings with clinicopathological data are lacking.

The main objective of this study was to evaluate the association between the antemortem hemostatic profile and the presence of fibrin deposition in postmortem tissues of horses with gastrointestinal disorders. Thus, hemostatic clinicopathological data (D‐dimer, prothrombin time, activated partial thromboplastin time, antithrombin) collected 0–24 h before death/euthanasia and postmortem tissue‐organ samples (kidney, lung, liver) from 48 horses with colic symptoms were retrospectively compared. Clinicopathological evidence of DIC was determined from available hemostatic data (≥3 abnormalities). Phosphotungstic acid hematoxylin (PTAH) and immunohistochemical (IMC) methods were used for histological examination. A fibrin score was established for each technique, tissue and horse, as well as the presence of histopathological DIC, defined as microthrombi present in ≥2 organs. The relationship was assessed by estimating odds ratios and 95% confidence intervals from logistic regression models.

Clinicopathological evidence of DIC was found in 11/48 horses whereas 9/48 (PTHA) and 7/48 (IMC) were considered to have histopathological DIC. No association was found between clinicopathological classification of DIC and postmortem classification based on tissue fibrin deposition. None of the measured hemostatic parameters was significantly different between horses with or without histopathological DIC.

In conclusion, routine hemostatic abnormalities do not seem to help in predicting histological evidence of DIC found at necropsy in horses with gastrointestinal disorders.

(This report represents a part of the thesis submitted by C. Cesarini to the Faculty of Veterinary Medicine, Universitat Autònoma de Barcelona, Spain, as required for the PhD degree.)

## 0007

### NSAIDS, DIARRHOEA AND BIG DATA

**M. Duz^1^, J. Marshall^2^, T. Parkin^1^**

^1^Boyd Orr Centre for Population and Ecosystem Health, School of Veterinary Medicine, College of Medical, Veterinary and Life Sciences, University of Glasgow, 464 Bearsden Road, Glasgow – G61 1QH, United Kingdom, ^2^Weipers Centre Equine Hospital, School of Veterinary Medicine, College of Medical, Veterinary and Life Sciences, University of Glasgow, 464 Bearsden Road, Glasgow – G61 1QH, United Kingdom

**Purpose of the study:** Evaluate whether recent NSAIDs, antimicrobial or corticosteroid treatment was more common in horses with diarrhoea (cases) than practice and date matched horses without diarrhoea (controls)

**Material and Methods:** This retrospective multicentre study used a database of electronic patient records (EPR) from 225,777 horses from North America. Using a validated text‐mining technique the database was searched for records reporting the clinical sign diarrhoea and for NSAIDs, antimicrobials and corticosteroids treatment. Cases and controls were compared using conditional logistic regression (significance: *P* < 0.05).

**Results:** Five thousand and six hundred eighteen cases of diarrhoea were identified and prevalence (±95%CIs) was determined (2.5%; 95%CIs: 2.4–2.6). Univariate analysis indicated recent NSAIDs (OR: 1.4; 95%CIs: 1.3–1.5; *P* > 0.001) or antimicrobial (OR: 1.5; 95%CIs: 1.4–1.7; *P* > 0.001) treatment or their combination (OR: 1.7; 95%CIs: 1.5–1.9; *P* > 0.001) were significantly more common in horses with diarrhoea. Recent corticosteroids treatment (OR: 0.6; 95%CIs: 0.5–0.7; *P* > 0.001) was more common in control horses. Horses with diarrhoea were more likely to have received flunixin meglumine (OR: 2.2; 95%CIs: 2.0–2.4; *P* < 0.001), but were less likely to have received phenylbutazone (OR: 0.6; 95%CIs: 0.5–0.7; *P* < 0.001). There did not appear to be an association between diarrhoea and recent firocoxib treatment (*P* = 0.21). Multivariable analysis indicated that recent treatment with flunixin meglumine (OR: 2.2; 95%CIs: 2.0–2.5) or antimicrobials (OR: 1.4; 95%CIs: 1.3–1.6) was associated with the development of diarrhoea. In contrast administration of corticosteroids was associated with a reduced likelihood of diarrhoea (OR: 0.5; 95%CIs: 0.4–0.6).

**Conclusion and practical application:** Diarrhoea is more common with NSAIDs and antimicrobials administration. This study demonstrates the value of automated text‐mining analysis to evaluate records from EPR systems not specifically designed for research purposes.

## 0008

### EVALUATION OF LIPASE AND AMYLASE LEVELS IN THE DIAGNOSTIC INVESTIGATION FOR THE EQUINE ACUTE ABDOMEN

**O. Kutasi^1^, L. Moravszki^1^, E.Bódai^1^, K. Joó^2^**

^1^Equine Department and Clinic, Faculty of Veterinary Sciences, Szent Istvan University, 2225‐H, Üllő, Dóra major, Hungary, ^2^Large Animal Research Group of the Hungarian Academy of Sciences and Szent István University, 2225‐H, Üllő, Dóra major, Hungary

Lipase and amylase enzymes are mainly produced by the pancreas in horses. Increased lipase and amylase levels occur with acute pancreatic damage. Perturbation of the pancreatic microvascular perfusion is an important pathogenic factor in cases of pancreatitis.

Our objective was to investigate the relationship between lipase and amylase levels and disease categories that cause acute abdominal pain in horses.

Medical records were reviewed for horses with acute colic between 2011 and 2013. Lipase and amylase levels were measured in 96 cases. Diagnosis was based on clinical signs, rectal examination, abdominal ultrasonography and when appropriate surgical or pathological findings. Using these diagnostic methods primary acute pancreatitis (AP) and proximal enteritis (PE) could not be differentiated in all cases.

Twenty‐five horses (26%) showed increased lipase or amylase values. Six of them (24%) was diagnosed with AP/PE. All horses with lipase level higher than 1000 IU/l belonged to this group. Six horses (24%) had right dorsal displacement (RDD) of the large colon with average lipase and amylase levels of 623.8 IU/l and 53.5 IU/l respectively and 13 cases had diverse causes of acute abdominal pain with average lipase level of 194.4 IU/l and amylase level of 13.5 IU/l, all with high standard deviations.

Fisher's exact test was used to verify that the lipase and amylase levels are useful in separating AP/PE from RDD or either of these from other diseases. The data suggest that extreme values could be very strong predictors, but larger sample would be needed for quantitative results in this direction.

## 0009

### Assessment of Cervical Oesophageal Motility by Transcutaneous Ultrasonography in Horses

**F. Malalana, S.J. Mack, M. Robin, C.M. McGowan**

Philip Leverhulme Equine Hospital, Leahurst, University of Liverpool, United Kingdom

Barium contrast function testing is considered the gold standard for assessment of oesophageal motility, whether by radiography or fluoroscopy; however, these techniques carry the risk of radiation exposure to personnel, and availability of fluoroscopy is limited. Endoscopic examination of the oesophagus is also commonly performed; whilst this technique is useful in the detection of anatomical abnormalities, it does not allow for the horse to be fed at the same time and the passage of the scope and the insufflation of air can alter oesophageal motility. Oesophageal manometry has also been described in horses but is rarely available. Sedatives, which are often necessary during these procedures, can also affect oesophageal peristalsis. The purpose of this study was to determine whether transcutaneous cervical oesophageal ultrasound represents a safe and easily available alternative for assessment of oesophageal motility.

Three horses presented for signs of abnormal oesophageal motility (recurrent episodes of oesophageal obstruction or persistent mucoid nasal discharge when head was lowered). Transcutaneous ultrasound examination of the cervical oesophagus was performed on the left side of the neck with a 5 MHz convex probe. Feeding of the horse whilst performing the examination allowed visualization of the progression of the food bolus in both cross section and longitudinal views. Well defined areas of abnormal motility, which were later confirmed by barium contrast studies, were detected in all 3 horses.

This study confirms that transcutaneous oesophageal ultrasound provides a safe, easy and readily available alternative to other techniques for assessment of cervical oesophageal motility in horses.

## 0010

### SHORT‐ AND LONG‐TERM SURVIVAL RATE OF HORSES AFTER LARGE INTESTINAL COLIC SURGERY – PRELIMINARY RESULTS

**M. Wehrli Eser^1^, R. Merk, P. Waldmeier**

Equine Department, Vetsuisse Faculty, University of Zurich, Zurich, Switzerland

There are few studies investigating outcomes after large intestinal colic surgery. The aim of this retrospective study was to describe the distribution of large intestinal surgical lesions and complications associated with colic surgery and to identify factors associated with short‐ and long‐term survival rates.

Medical records from 530 large intestinal surgical colic patients were reviewed. Type of lesion, complications, clinical and laboratory variables, short‐term (survival to discharge) and long‐term survival (1 and 5 years) were recorded. Fisher's exact test was used to assess the association of variables with survival rate.

Diagnoses were colon torsion (33%), right dorsal displacement of the large colon (RDD, 13%), retroflexion of the colon (RFC, 11%), pelvic flexure impaction (9%), left dorsal displacement (LDD, 5%), intestinal distension (5%), and various other problems (24%). 456 horses (86%) recovered from surgery. Common post‐surgical complications included colic (13%), incisional complications (13%), jugular thrombophlebitis (11%) and colitis (11%). Survival rate to discharge was 72% (384/530). After 1 and 5 years, respectively, 92% (170/184, 200 lost follow‐up) and 70% (64/91, 293 lost) of horses were still alive. Variables significantly associated with increased short‐term survival were no or mild abdominal pain, heart rate between 28–48/min and normal to mildly changed mucous membrane color. PCV > 46% had a negative influence on short term and 1‐year survival. Intraoperative diagnosis, duration of surgery, and intraoperative contamination significantly influenced short‐term survival. Short‐term survival rate was highest with LDD (100%), RFC (93%) and RDD (86%).

The short‐ and long‐term survival rates are comparable to other studies.

## 0011

### COMPARISON OF BODY WEIGHT LOSS IN SURVIVING AND NON‐SURVIVING CASES OF CHRONIC EQUINE GRASS SICKNESS

**R.C. Jago, I. Handel, C.N. Hahn, B.E. Waggett, R.S. Pirie, J.A. Keen, B.C. McGorum**

The Royal Dick School of Veterinary Studies and Roslin Institute, The University of Edinburgh, Easter Bush Campus, Midlothian, United Kingdom

Equine grass sickness is a multiple system neuropathy primarily causing degeneration of autonomic and enteric neurons. While the acute and sub‐acute forms of the disease are invariably fatal, *some* chronic grass sickness (CGS) cases survive. Unfortunately, objective criteria for predicting survival of CGS cases are currently lacking. The main aim of this study is to determine whether the rate and magnitude of body weight loss can be used as an objective predictor of survival.

A single centre retrospective observational study was performed. Case records of all horses admitted to the Dick Vet Equine Hospital between 1998 and 2013 for management of CGS were analysed. Signalment, survival status, indication(s) for euthanasia, duration of disease at hospitalisation, and body weights were analysed, and data for survivors and non‐survivors compared.

Two hundred and fourteen horses were included in the study, including 114 survivors to hospital discharge and 100 non‐survivors. A non‐linear model was fitted to each horse's weight records. Using parameters from the model we estimated the initial mean weight loss in non‐surviving horses to be 0.85% per day and 0.59% in survivors. By the 30th day of disease the mean daily weight loss had increased to 2.76% in non‐survivors and was negligible in survivors. Further analysis of the data using a classification model suggested that weight records of individual horses over the first 25 days of disease may be moderately predictive of outcome; however this is most likely to be of limited benefit in the individual clinical case setting.

## 0012

### THE EFFECT OF A NEWLY DESIGNED PROBIOTIC ON PREVENTION OF EQUINE NEONATAL DIARRHEA

**A. Schoster^1^, M. Abrahams^2^, H.R. Staempfli ^2^, J.S. Weese^3^, L. Guardabassi^4^.**

^1^Equine Department, Vetsuisse Faculty, University of Zurich, Winterthurerstrasse 260, 8057 Zurich, Switzerland, ^2^Department of Clinical Studies, Ontario Veterinary College, University of Guelph, N1G2W1 Guelph, ON, Canada, ^3^Department of Pathobiology, Ontario Veterinary College, University of Guelph, N1G2W1 Guelph, ON, Canada, ^4^Department of Veterinary Disease Biology, Faculty for Health and Medical Sciences, University of Copenhagen, Gronnegardsvej 15, 1870 Frederiksberg C, Denmark

Up to 60% of foals develop diarrhea within 6 months after birth. Preventative measures are limited but probiotics could be used. The objective was to evaluate the effect of a newly designed probiotic on the incidence of neonatal foal diarrhea.

*Lactobacillus rhamnosus LHR 19 and SP1, L. plantarum LPAL and BG112* and *Bifidobacterium animalis lactis* combined were evaluated in a 4‐week randomized placebo controlled field trial in 53 neonatal foals. Foals were monitored for fecal consistency, depression, suckle reflex, fever and veterinary treatments. The Chi‐square‐, Mann Whitney Test and ANOVA were used to compare the incidence and duration of diarrhea, soft feces, and diarrhea plus clinical signs or veterinary treatment between treatment and age groups.

The incidence of diarrhea was 30/53 (57%). Neither incidence of diarrhea (*P* = 0.69) nor soft feces (*P* = 0.85) or both (*P* = 0.26) were statistically significant between groups. Incidence of diarrhea concurrent with clinical signs and diarrhea requiring treatment were not statistically different between groups (*P* = 0.32, *P* = 0.33 respectively). Duration of diarrhea, soft feces or both was not significantly different between groups (*P* = 0.28, *P* = 0.84, *P* = 0.26 respectively). Age had a significant effect on incidence and duration of diarrhea. There was a trend towards increased diarrhea occurrence in probiotic treated foals (*P* = 0.08).

There was no benefit of administering probiotics, and even a potential trend for adverse effects. Whether the choice of strains or the dose was inadequate is unknown. Incidence of diarrhea was highest between 8–14 days after birth. Microbiological cultures will be performed to assess the effect on pathogen shedding.

## 0013

### ADMISSION SEVERE HYPONATRAEMIA IN HOSPITALISED FOALS

**N.M. Collins^1^, J.E. Axon^1^, J.B. Carrick^1^, C. Russell^2^, P. Dow^1^, J.E. Palmer^2^**

^1^Scone Equine Hospital, Scone, Australia, ^2^University of Pennsylvania, Kenneth Square, USA

**Purpose of the study:** To establish the prevalence of severe hyponatraemia in hospitalised foals on admission to an ICU and to document the diagnosis, treatment and outcome of these foals.

**Methods:** Records from foals aged <3 months presenting to the ICU (2002–2012) were reviewed and foals with severe hyponatraemia (serum sodium<122 mmol/l) on admission laboratory data were identified and further evaluated. Data analysed included signalment, clinical and laboratory parameters, diagnosis, treatment and outcome.

**Results:** Admission severe hyponatraemia was identified in 4% of foals (69/1718). Median admission serum sodium level was 116 mmol/l (IQR = 109.5–120, *n* = 69). Eleven of the 69 foals (15.9%) presented with neurological signs attributable to hyponatraemic encephalopathy. The three most common primary diagnoses were renal disease [18(26.1%)], enterocolitis [16(23.2%)] and uroperitoneum [15(21.7%)]. Treatment consisted of therapy aimed at the primary disease and correction of the hyponatraemia. Fifty of the 69 foals (72.5%) with admission severe hyponatraemia survived to hospital discharge and 42 of these 50 foals (84%) survived at least 4 months after discharge.

**Conclusions:** The prevalence of admission severe hyponatraemia in this study population was 4%. The majority of foals with severe hyponatraemia did not demonstrate direct clinical manifestations as a result of the low serum sodium level. However, hyponatraemic encephalopathy should be considered in the differential diagnoses for foals showing abnormal neurological signs such as marked depression and seizures.

## 0014

### EFFECT OF COMMONLY USED ELECTRODE CONFIGURATIONS ON ELECTROCARDIOGRAPHIC PARAMETERS IN THE STANDING HORSE: PRELIMINARY RESULTS

**D. De Clercq, B. Broux, S. Claasen, N. Van Der Vekens, S. Ven, S. Sys, A. Decloedt, G. vanLoon**

Department of Large Animal Internal Medicine, Ghent University, Belgium

Electrocardiography (ECG) is used for determination of the heart rate, regularity and the effects of drugs or disease on the electrical activity of the heart. Usually electrodes are positioned along the mean electrical axis but no universal method for recording an ECG in horses is described. To determine the effect of electrode position on amplitude and duration of the different waves; ECGs were recorded from 8 different commonly used electrode positions in 6 standing non‐sedated healthy horses. In each configuration, P and QRS amplitude and P, QRS and QT duration were measured from 20 cycles, heart rate was between 35–45 beats per minute.

The coefficient of variation between configurations for P and QRS amplitude was 31 and 36%, and for P, QRS and QT duration 24, 9 and 2%, respectively. A significantly higher P amplitude was obtained with the negative electrode on the right side of the withers and the positive electrode 15 cm behind and above the left olecranon. A significantly higher QRS amplitude and longer P and QRS duration was obtained with the negative electrode in the lower third of the right jugular vein and the positive electrode on the apex of the heart. A significantly longer QT duration was obtained with the negative electrode on the manubrium sterni and the positive electrode on the xiphoid.

The electrode position markedly influences both amplitude and duration of ECG waves. Clinical and scientific ECG indication should therefore dictate recording lead choice and lead‐specific reference values should be used.

## 0015

### SOTALOL HYDROCHLORIDE AS AN ORAL ANTIARRHYTHMIC IN THE HORSE?

**B. Broux^1^, N. Van Der Vekens^1^, A. Decloedt ^1^, D. De Clercq^1^, S. Croubels^2^, G. vanLoon^1^**

^1^Department of Large Animal Internal Medicine, Faculty of Veterinary Medicine, Ghent University, Merelbeke, Belgium, ^2^Department of Pharmacology, Toxicology and Biochemistry, Faculty of Veterinary Medicine, Ghent University, Merelbeke, Belgium

**Introduction:** Arrhythmias are common in horses. Some, such as frequent atrial or ventricular premature beats, may require long‐term anti‐arrhythmic therapy. However, there is currently little information on anti‐arrhythmic drugs for chronic oral use in horses. In humans and small animals, sotalol hydrochloride is often used for chronic oral anti‐arrhythmic therapy. Sotalol prolongs repolarisation and the effective refractory period in all cardiac tissues^1,2^. Sotalol pharmacokinetics in men show an excellent oral bioavailability and permit long dosing intervals^1–4^. No information on pharmacokinetics or pharmacodynamics is available in horses.

**Material and methods:** In a cross‐over design, six starved horses received 1 mg/kg sotalol intravenously and orally, with a four week washout period in between. Two horses with access to feed were given 2 mg/kg sotalol hydrochloride twice daily for five days. Plasma sotalol levels were determined at different time points and horses were under ECG‐surveillance.

**Results**: Oral bioavailability was 48 ± 15%. When a 1 mg/kg dose was given orally, peak plasma concentrations of 316.53 ± 70.44 ng/mL(Cmax) were reached after 0.94 ± 0.43 hours(Tmax). When administered with food, bioavailability was delayed and slightly decreased, reaching plasma concentrations between 139 and 313 ng/mL. There was no significant decrease in heart rate or prolongation in QT interval. Adverse effects of sweating (*n* = 3) and mild colic signs (*n* = 1) were only seen after intravenous administration.

**Discussion:** Because of its good oral bioavailability sotalol is a potentially useful anti‐arrhytmic oral drug for horses. This dose appeared safe with only mild, temporary side‐effects. Additional pharmacokinetic and pharmacodynamic studies are needed to establish correct dosing.

## 0016

### HEMOCHROMATOSIS AND LIVER FAILURE IN 11 HORSES DUE TO CHRONIC IRONINTOXICATION

**M.J.P. Theelen^1^, M. Beukers^2^, G.C.M. Grinwis^3^, M.M. Sloet van Oldruitenborgh‐Oosterbaan^1^**

^1^Department of Equine Sciences – Internal Medicine, Faculty of Veterinary Medicine, Utrecht University, Yalelaan 114, 3584 CM, Utrecht, the Netherlands, ^2^Department of Clinical Sciences of Companion Animals – Diagnostic Imaging, Faculty of Veterinary Medicine, Utrecht University, Yalelaan 108, 3584 CM, Utrecht, the Netherlands, ^3^Department of Pathobiology – Veterinary Pathology Diagnostic Centre, Faculty of Veterinary Medicine, Utrecht University, Yalelaan 1, 3584 CL, Utrecht, the Netherlands

Iron toxicosis is rarely reported in horses and chronic excessive oral iron intake is thought not to cause any clinical symptoms in horses. This case series describes 11 genetically unrelated horses with evidence of liver damage due to chronic intake of excessive amounts of iron.

The first case presented with hepatic encephalopathy. At necropsy, excessive iron accumulation was found in several organs including the liver (hemochromatosis). Further examination of all horses on the same farm showed poor body condition scores and rough hair coats. Serum iron was increased in ten horses (mean of all horses 73.3 ± 15.6 μmol/l; normal 18–54 μmol/l) and iron saturation levels were increased in all horses (mean 90.7 ± 2.6%; normal 40%). Ten horses had elevated GGT (mean of all horses 910 ± 635 IU/l; normal <34 IU/l) and nine horses had increased bile acids (mean of all horses 29 ± 23 μmol/l; normal <11.5 μmol/l). Other elevated parameters in affected horses were total iron binding capacity, AF, AST, LDH, ammonia, WBC, TP and beta‐2‐globulins. Ultrasonographic evaluation of the liver of five horses showed in all five enlargement of the liver with increased echogenicity and histologic evaluation of liver biopsies showed severe hemochromatosis and cirrhosis. Water, soil and feedstuff were analysed for iron content: water iron level 72.5 mg/l (acceptable <0.5 mg/l), iron levels in soil and feedstuff were not increased.

It was concluded that the drinking water (ditch) was the source of the excessive iron intake and this study indicates that chronic excessive iron intake can lead to hemochromatosis and liver failure.

## 0017

### TETANUS IMMUNITY IN HORSES IN NORTHERN GERMANY

**A. Bonetto, J.‐M.V. Cavalleri, B. Ohnesorge**

Clinic for Horses, University of Veterinary Medicine, Bünteweg 9, 30559 Hannover, Germany

We aimed to characterize tetanus immunity in a population of adult horses in relation to their vaccination status. A convenience sample of 535 horses (2–29 years) presented at the Clinic for Horses was considered. Antibody titer against *Cl. tetani* was evaluated through immunochromatography (Fassisi TetaCheck^®^); the vaccination history as reported in the passport was recorded and assigned to one of the following groups:

- A (31.5%): Ground immunization and regular boosters q 2 years
- B (10.3%): Ground immunization, irregular boosters
- C (12.4%): Vaccinations at least every 2 years for more than 50% of the lifespan, no ground immunization
- D (10.5%): Irregular vaccinations (intervals > 2 years)
- E (35.3%): No passport or record available.

Data were matched through statistical analysis (ANOVA, χ^2^, Fisher′s exact test as appropriate). There was a statistically significant (*P* < 0.0001) correlation between a titer of more than 1 IU/mL and old age (>20 years), and with more than 10 tetanus vaccinations in the horse′s life.

Groups A, B, C showed a solid protective immunity (negatives <3%) whereas in groups D and E higher percentages of failed immunization (12.3% and 12.7%, respectively) were found. In an alarming amount of patients (35.3%), no vaccination record was available.

We demonstrated that regular vaccination protocols yield satisfactory results; we suggest that yearly boosters may not be required throughout the horse′s life after ground immunization is achieved. Group E′s high percentage (12.7%) of negative titers (<0.01 IU/mL) stresses the importance of accurate keeping of equine vaccination records.

## 0018

### THE EFFECT OF THE TOPICAL APPLICATION OF DELTAMETHRIN TO HORSES ON BLOOD‐FEEDING BY *CULICOIDES* MIDGES

**M. Robin^1^, C. McGowan^2^, D. Archer^1^, C. Garros^3^, L. Gardes^3^, M. Baylis^1^**

^1^Department of Epidemiology and Population Health, Institute of Infection and Global Health, University of Liverpool, Leahurst, Chester High Road, Neston, Cheshire CH64 7TE, United Kingdom, ^2^Institute of Ageing and Chronic Disease, Faculty of Health and Life Sciences, University of Liverpool, Leahurst, Chester High Road, Neston, Cheshire CH64 7TE, United Kingdom, ^3^Cirad, UMR15 CMAEE; INRA UMR1309 CMAEE, Montpellier, France

African horse sickness (AHS) is a vector‐borne disease spread by *Culicoides* midges. In the UK, DEFRA currently suggests the use of topical deltamethrin for AHS control; however, no data is available regarding its efficacy in the horse. Northern European *Culicoides* of the Obsoletus and Pulicaris Groups may be able to act as AHS vectors, based on their ability to transmit the similar disease, bluetongue. Studies in mainland Europe have demonstrated that these species are abundant around equids. The aims of this study were to investigate the effect of topical deltamethrin on the blood‐feeding rate of *Culicoides* on horses and to investigate which species appear to preferentially blood‐feed on horses.

Three pairs of horses were placed in partially enclosed cages, which allowed samples representing the *Culicoides* interacting with each individual horse to be sampled over 1 hr. Four sessions were run before 1 horse from each pair was treated with 1% topical deltamethrin and another 4 sessions were completed. The species of each midge collected was determined by a combination of light microscopy or PCR and each midge examined to see if it had blood‐fed.

There was no significant treatment effect of topical deltamethrin on the blood‐feeding of *Culicoides* on horses. The most abundant species collected were members of the Obsoletus (44.3%) and Pulicaris (34.7%) Groups. There was a significant relationship between blood‐feeding and species, with *Culicoides* from these 2 groups more likely to have blood fed.

The results do not support the use of topical deltamethrin as a method for minimizing the spread of AHS. Obsoletus and Pulicaris Group *Culicoides* were the most likely to blood‐feed on horses, supporting their potential role as vectors if AHS virus were to reach the UK.

## 0019

### FIBROSIS IN WHITE ADIPOSE TISSUE OF HORSES WITH EQUINE METABOLIC SYNDROME AND PITUITARY PARS INTERMEDIA DYSFUNCTION

**T.M. Fordham^1^, R.A. Morgan^1,2^, P.W. Hadoke^2^, B.W. Walker^2^, J.A. Keen^1^**

^1^Royal Dick School of Veterinary Studies, University of Edinburgh, Easter Bush Campus, Midlothian EH25 9RG, United Kingdom, ^2^BHF Centre for Cardiovascular Science, The Queen's Medical Research Institute, University of Edinburgh, 47 Little France Crescent, Edinburgh EH16 4TJ, United Kingdom

Fibrosis of white adipose tissue (WAT) is a hallmark of pathology in humans with metabolic syndrome. Fibrosis indicates hypoxia within cells leading to the attraction and retention of pro‐inflammatory cytokines implicated in the development of insulin resistance. A pro‐inflammatory state may result in activation of the hypothalamus‐pituitary‐adrenal axis resulting in derangements in hormones such as ACTH and cortisol. This study investigated the role of adipose fibrosis in horses with equine metabolic syndrome (EMS) and pituitary pars intermedia dysfunction (PPID).

Samples of peri‐renal adipose tissue were obtained at post‐mortem from horses without disease (*n* = 4), with EMS (*n* = 4) and with PPID (*n* = 6). The samples were paraffin embedded, sectioned and stained with haematoxylin‐eosin for evaluation of the structure and picrosirius‐red (PSR) to identify collagen. Image Pro software was used to quantify PSR staining; ten areas were selected by computer program at random and the adipocyte size measured by an observer blinded to the group. The number of blood vessels within a designated area was quantified by a blinded observer. There was increased fibrosis in horses with EMS (13.87 ± 5.70) and PPID (20.43 ± 15.39) versus healthy controls (5.26 ± 2.97) but this did not reach statistical significance (*P* = 0.06). Adipocyte size and vessel number were not different between the groups. Vessel number was negatively correlated with body condition score (*P* = 0.02). This study suggests that EMS and PPID may be associated with abnormal adipose tissue. Further work is necessary to further elucidate the role of WAT in equine endocrine disease.

## 0020

### PREDICTING LAMINITIS RISK IN HORSES WITH ENDOCRINE DISEASE

**R. A. Morgan^1,2^, P. W. Hadoke^1^, B. W. Walker^1^, J. A. Keen^2^**

^1^BHF Centre for Cardiovascular Science, The Queen's Medical Research Institute, University of Edinburgh, 47 Little France Crescent, Edinburgh EH16 4TJ, United Kingdom, ^2^Royal Dick School of Veterinary Studies, University of Edinburgh, Easter Bush Campus, Midlothian EH25 9RG, United Kingdom

The majority of cases of laminitis result from endocrine disease, either Pituitary Pars Intermedia Dysfunction(PPID) or Equine Metabolic Syndrome(EMS). However, horses with these diseases do not always develop laminitis and identifying those most at risk is a challenge. This study aimed to investigate clinical and mechanistic factors which predict laminitis. Horses with no signs of systemic inflammation, destined for euthanasia were eligible for inclusion. Prior to euthanasia a history, full clinical examination and venous blood were obtained. Serum insulin and plasma ACTH, cortisol and αMSH concentrations were determined. At post‐mortem adipose tissue (peri‐renal, linea alba and neck crest) and pituitary glands were collected. RNA was extracted from adipose tissue and transcript levels of 11β‐hydroxysteroid dehydrogenase1(11β‐HSD1) quantified. The pituitary glands were scored histologically. The hooves were sectioned and examined.

Fifteen horses with no evidence of endocrinopathic laminitis and 22 with endocrinopathic laminitis were recruited. A binary logistic regression analysis was used to determine factors associated with the presence of laminitis. At a univariable level factors retained were increased insulin, BCS and RNA transcript levels of 11β‐HSD1. In a multivariable model only insulin (OR 1.09,CI 1.01–1.16, *P* = 0.02) and BCS (OR 2.64,CI 1.01–6.88, *P* = 0.047) remained in the model (LLR ‐12.5, G = 21.029, *P* < 0.0001).

This study indicates that insulin and adiposity are the best predictors of laminitis risk in this mixed cohort. This suggests that insulin dysregulation is the common feature of both EMS and PPID that results in laminitis and that assessment of insulin status is essential in all cases of laminitis.

## 0021

### EQUINE PROCALCITONIN AS A POTENTIAL INFLAMMATORY BIOMARKER IN HORSES

**D. Teschner^1^, A. Barton^1^, C. Koopmann ^1^, M. Rieger^2^, H. Gehlen^1^**

^1^Equine Clinic, Free University of Berlin, Oertzenweg 19b, 14163 Berlin, Germany, ^2^Research Unit Microbe‐Plant Interactions, Helmholtz Zentrum München, Ingolstädter Landstr. 1, 85764 Neuherberg, Germany

In human medicine, procalcitonin (PCT) is accepted as a highly specific and early marker for microbial infections and sepsis. The aim of this study was to compare plasma PCT concentrations in horses questionable of sepsis to healthy individuals using a newly validated sandwich‐ELISA for the detection of equine PCT. Plasma samples of 24 healthy horses and five horses with clinical signs of sepsis were tested. All horses were classified according to a sepsis‐score adapted from Breuer et al. (2012) including data from clinical examination and laboratory data at the time of first presentation as positive for sepsis (group A, ≥ 7 score points), questionable (group B, 4–6 score points), or negative for sepsis (group C, ≤ 3 score points).

The five horses with clinical signs of sepsis were assigned to group A (2 mares, 3 geldings, age: 15 ± 4 years, bodyweight: 520 ± 68 kg), 4 healthy horses (3 geldings and 1 mare, age: 14 ± 5 years, bodyweight: 575 ± 86 kg) were classified as group B and 20 healthy horses (14 geldings and 6 mares; age: 13 ± 5 years, BDW: 529 ± 58 kg) were classified as group C. We found a significantly higher median ePCT concentration of 8450 ng/mL in the sepsis group in contrast to 47 ng/mL for the control groups B and C (*P* = 0.0006).

All plasma samples of the sepsis group showed increased ePCT indicating the relevance of ePCT as a valuable sepsis marker in horses.

## 0022

### EFFECTS OF DIFFERENT CATECHOLAMINES ON MYOCARDIAL FUNCTION IN ISOFLURANE ANAESTHETIZED HORSES

**C. Hopster‐Iversen, K. Hopster, S. B. R. Kästner, P. Stadler**

Equine Clinic, University of Veterinary Medicine Hanover, Foundation, Bünteweg 17, 30559 Hanover, Germany

Aim of the study was to assess the effects of dopamine, dobutamine, noradrenaline and phenylephrine on the myocardial function in anaesthetized horses.

Six warmblood horses without cardiac diseases were premedicated with xylazine (0.8 mg/kg); anaesthesia was induced with midazolam/ketamine and maintained with isoflurane (1.4%). After 60 minutes of equilibration dopamine (5 μg/kg/min), dobutamine (3 μg/kg/min), noradrenaline (0.5 μg/kg/min) and phenylephrine (3 μg/kg/min) were randomly administered for 15 minutes with a washout between infusions of 45 minutes. Myocardial function was measured by fractional shortening (FS%) and myocardial velocities (E‐wave, A‐wave, systole) in the interventricular septum and left‐ventricular free wall. Ultrasound was performed at rest, after sedation, before and during catecholamine infusion. Cardiac output (CO) was measured by thermodilution before and during catecholamine infusion. An ANOVA for repeated measurements and Tukey′s post hoc test were used (alpha = 5%).

Anaesthesia resulted in a significant decrease of FS, systolic and early diastolic myocardial velocity. Dobutamine increased FS, CO and systolic myocardial velocity in comparison to baseline values in anaesthesia. A significant decrease of FS, systolic and early diastolic myocardial velocities were observed after administration of noradrenaline and phenylephrine, respectively when compared to resting values in the awake horses. Phenylephrine also decreased CO compared to baseline values in anaesthesia. At the tested dose rates only dobutamine and dopamine increased CO during anaesthesia.

The present study demonstrated a significant influence of anaesthesia and different catecholamines on myocardial function.

## 0023

### EXERCISE INDUSED PULMONARY HAMORRHAGE (EIPH) IN TROTTERS: IMPACT ON LATER PERFORMANCE

**C. F. Ihler, S. H. Olsen**

Norwegian University of Life Sciences, Oslo, Norway

In a previous study by the same authors (unpublished) the prevalence of EIPH in Standardbreds (ST) and Norwegian Coldblooded Trotters (NCT) was found to be 66.9 (101/151) and 36.1%(51/141), respectively, using the grading 0–4 (Hinchcliff et al., 2005). Forty‐seven bleeders were followed up by new post‐race tracheoscopy; Forty‐two bled repeatedly. The purpose of the present study was to evaluate the performance in bleeders and none‐bleeders in both breeds throughout their careers. The variables used were prize money, prize money per start, length of career (months) and number of starts after the initial evaluation. Data were obtained from The Norwegian Trotters Association. The variables were compared using the non‐parametric Wilcoxon/Kruskal‐Wallis test.

The statistical analysis revealed no statistical differences (*P* < 0.05) for any of the performance variables in the two breeds. When comparing individuals showing grade 3 and 4 at the initial examination to grade <3. The NCTs showed decreased performance measured as length of career (*P* = 0.04) and prize money per start (*P* = 0.03). In STs, there was no difference between individuals with EIPH grades 3 and 4 and grade <3. In STs, it can be concluded that EIPH have no impact on future performance and might be considered as a normal response to race as two thirds of horses show EIPH. In NCTs, EIPH grades 3 and 4 have impact on length of career and prize money per start.

## 0024

### THE EFFECT OF FEEDING ON THE REPEATABILITY OF RIGHT DORSAL COLONIC WALL THICKNESS MEASUREMENT

**N. Kerbyson^1^, M. Duz^1^, M. Cathcart^2^, K. Hughes^3^, T. D. H. Parkin^1^, S. Love^1^**

^1^University of Glasgow, School of Veterinary Medicine, 464 Bearsden Rd, Glasgow G61 1QH, United Kingdom^2^University of Adelaide, Adelaide, South Australia, ^3^Charles Sturt Univeristy, Boorooma St, North Wagga, NSW, Australia

The inter‐operator repeatability of ultrasonographic colonic wall thickness measurement has been assessed (Bithell et al., 2010) however it is not known if recent feeding alters this measurement. The aim of this study was to assess if recent feeding affected the repeatability of RDC wall thickness measurements and to assess the inter‐operator repeatability of these measurements.

Nine healthy horses underwent ultrasonographic evaluation of the RDC by two experienced operators on two consecutive days; the horse was fasted for 16 hours prior to examination on the first day and given free access to food on the second. The colonic wall thickness was measured immediately axial and ventral to the liver and in proximity to the right ventral colon at the 10th‐15th intercostal space inclusively: up to 12 measurements were obtained for each horse. Bland Altman plots were used to assess inter‐operator agreement and agreement of measurements in fed and fasted horses.

The RDC could be visualised most consistently in the 11th or 12th intercostal space with all attempts at measurement successful. Interoperator agreement was good with a bias of 0.07 mm between operators (95% confidence interval: −2.16 to 2.02 mm. There was a mean difference of +0.18 mm (fasted> fed) between fed and fasted horses (95% confidence intervals: −1.57 to +1.22 mm).

This study shows that RDC wall thickness measurement is highly variable. Fasting is associated with a change in wall thickness although the direction of this change is inconsistent. The feeding status of the horse should be considered when performing repeated measurements of right dorsal colonic wall thickness.

## 0025

### ULTRASTRUCTURAL MITOCHONDRIAL ALTERATIONS IN EQUINE MYOPATHIES OF UNKNOWN ORIGIN

**K. Van Driessche^1^, K. Chiers^1^, R. Ducatelle^2^, R. Van Coster^2^, J. H. Van Der Kolk^3,4^**

^1^Laboratory of Veterinary Pathology, Department of Pathology, Bacteriology and Avian Medicine, Faculty of Veterinary Medicine, Ghent University, Salisburylaan 133, 9820 Merelbeke, Belgium, ^2^Division of Child Neurology and Metabolism, and Neuromuscular Reference Center, Department of Pediatrics and Genetics, Faculty of Medicine, Ghent University, De Pintelaan 185, 9000 Gent, Belgium, ^3^Euregio Laboratory Services, section Equine Metabolic and Genetic Diseases, Stadionplein 46, 6225 XW Maastricht, the Netherlands, ^4^Division of Clinical Veterinary Medicine, Swiss Institute for Equine Medicine ISME, Vetsuisse Faculty, ALP Haras, University of Bern, Länggassstraβe 124, 3012 Bern, Switzerland

**Purpose of the study:** The purpose of the study was to examine the ultrastructure of muscle mitochondria in equine cases of myopathy of unknown origin in an attempt to disclose potential new mitochondriopathies.

**Methods:** Biopsies of *vastus lateralis* of the *Musculus quadriceps femoris* were taken predominantly immediately post mortem and processed for transmission electron microscopy. As a result, electron micrographs of 90 horses in total were available for analysis comprising 4 control horses, 16 horses suffering from myopathy, and 70 otherwise diseased horses.

**Results:** Following a thorough clinical and laboratory work‐up, four out of five patients that did not fit into the usual algorithm to detect known causes of myopathy showed ultrastructural mitochondrial alterations resembling similar mitochondrial myopathies as described in humans. Small mitochondria with zones with complete disruption of cristae associated with lactic acidemia were detected in a 17‐year‐old pony mare, extremely long and slender mitochondria with longitudinal cristae in a 5‐year‐old Quarter horse stallion, a mixture of irregular extremely large mitochondria (measuring 2500 by 800 nm) next to smaller ones in a 8‐year‐old Hannovarian mare, and round mitochondria with only few cristae in a 11‐year‐old pony gelding. It remains uncertain whether the subsarcolemmal mitochondrial accumulations observed in the fifth patient have any pathological significance.

**Conclusions:** Ultrastructural evidence was found for at least four potential new mitochondriopathies in horses.

**Practical applications:** The possibility of a mitochondriopathy should be included in the differential diagnosis of muscle weakness and electron microscopy of muscle tissue should be considered as a valuable diagnostic tool.

## 0026

### KAOLIN ACTIVATED THROMBOELASTOGRAPHY IN 20 HEALTHY HORSES

**K. Machackova, M. Boselova, I. Uhrikova, J. Doubek**

Department of Physiology, Faculty of Veterinary Medicine, University of Veterinary and Pharmaceutical Sciences Brno, Palackeho 1/3, Brno, 612 42, Czech Republic

In human medicine thrombohemorrhagic disorders as a severe complication of acute illness are diagnosed not only by routine coagulation tests but also by thromboelastography (TEG). Examination is performed in whole blood and thus evaluates both cellular and soluble components of hemostasis and fibrinolysis. The aim of this study was to define physiological values of kaolin activated thromboelastography in horses.

Blood from 20 healthy horses was collected into citrated tubes. One milliliter from each tube was used for TEG (R‐time, K‐time, α‐angle, MA and LY30 were measured), rest of the blood was centrifuged (3000 rpm, 15 min) and routine coagulation tests: PLT, PT, aPTT, fibrinogen (FBG) and D‐dimers (DD) were performed to compare with TEG. Samples were stored in laboratory temperature and measured within after 2 hours after collection.

Thromboelastography results, median/min‐max values shown: R (min) 11.1/3.2–17.4, K (min) 3.10/1.5–5.2, α (°) 52.30/28.7–67.5, MA (mm) 55.3/42.7–66.7, LY30 (%) 0.4/0–3.4. Routine coagulation tests results were following (median/min‐max values shown): PLT (*10^9^/L) 124/89–192, PT (s) 16.0/11.6–19.7, aPTT (s) 60.8/34.7–92.1, FBG (g/L) 1.85/0.36–5.16, DD (mg/L) 0/0–0.4.

Based on this study, preliminary physiologic values of kaolin activated TEG are following R (min) 8–15, K (min) 2.5–5, α (°) 43–58, MA (mm) 48–57, LY30 (%) 0–1. Routine coagulation tests were different from ranges of our laboratory and were adjusted to actual results to be used in further research: PLT (*10^9^/L) 100–200, PT (s): 14–18, aPTT (s) 54–80, FBG (g/L) 1.5–4.5, DD (mg/L) 0–0.1.

Kaolin activated thromboelastography can be useful diagnostic tool for monitoring coagulation and fibrinolysis.

## 0027

### HDL, LDL AND TOTAL CHOLESTEROL IN HEALTHY AND SEPTIC NEONATAL FOALS

**J. Mariella, F. Freccero, A. Lanci, F. Dondi, L. Taddei, C. Castagnetti**

Department of Veterinary Medical Sciences, University of Bologna, Via Tolara di Sopra 50, 40064 Ozzano dell'Emilia BO, Italy

Total cholesterol (TC), HDL‐ and LDL‐cholesterol have never been investigated in neonatal foals. The aims of this study were: a) to measure serum TC, HDL‐ and LDL‐cholesterol in healthy foals at birth (T0) and after 72 hours (T72), and in septic foals at admission, b) to evaluate if any difference exists between healthy and septic foals and between surviving and non‐surviving septic foals.

Twenty‐five foals ≤3 day‐old were included: 13 healthy and 12 septic foals (mean age 24 ± 18 hours), on the basis of positive blood culture and systemic inflammatory response. TC, HDL‐ and LDL‐cholesterol were measured with colorimetric methods. Data were analyzed with paired and independent t‐test, or 1‐way ANOVA, and reported as mean ± SD.

In healthy foals, TC, HDL‐ and LDL‐cholesterol at T0 (TC 143 ± 28 mg/dL; HDL‐ 31 ± 12 mg/dL; LDL‐cholesterol 101 ± 27 mg/dL) were significantly lower than at T72 (TC 185 ± 49 mg/dL; HDL‐ 50 ± 6 mg/dL; LDL‐cholesterol 124 ± 43 mg/dL) (*P* < 0.01, *P* < 0.001, and *P* < 0.05, respectively). TC, HDL‐ and LDL‐cholesterol in septic foals at admission (TC 296 ± 95 mg/dL; HDL‐ 45 ± 12 mg/dL; LDL‐cholesterol 219 ± 80 mg/dL) were significantly higher than in healthy foals at T0 and T72 (*P* < 0.0001). There were no differences between surviving and non‐surviving septic foals.

TC, HDL‐ and LDL‐cholesterol increase in healthy foals at birth and in septic foals at admission could represent a derangement of lipid metabolism. Also the increase in serum amyloid A, the dominant HDL apoprotein during the acute phase response, could influence HDL‐cholesterol.

## 0028

### GRAZING HORSES ARE EXPOSED TO TERRESTRIAL CYANOBACTERIA

**B. C. McGorum^1^, J. S. Metcalf^2^, S. A. Banack^2^, G. A. Codd^3^**

^1^Royal Dick School of Veterinary Studies and Roslin Institute, University of Edinburgh, Roslin, EH25 9RG, Scotland, UnitedKingdom, ^2^The Institute for Ethnomedicine, 240 East Deloney Avenue, Jackson WY 83001‐3464, USA, ^3^Biological and Environmental Sciences, University of Stirling, Stirling, FK9 4LA, United Kingdom

While toxins from aquatic cyanobacteria are a well‐recognised cause of disease in birds and animals, exposure of grazing livestock to terrestrial cyanobacteria has not been previously described. In this study, terrestrial cyanobacteria, predominantly *Phormidium* spp., were identified in the biofilm of plants from most horse fields investigated. The density of *Phormidium* filaments varied markedly, both within and between fields. Lower numbers of other cyanobacteria and microalgae (diatoms, *Closterium*) were also present on many plants. Further work was performed to test the hypothesis that ingestion of cyanobacterial hepato‐ and neurotoxins contributes to the pathogenesis of some currently unexplained diseases of grazing horses, including equine grass sickness (EGS), equine motor neuron disease (EMND) and hepatopathy. *Phormidium* population density was significantly higher on EGS fields than on control fields. The cyanobacterial neurotoxin 2,4‐diaminobutyrate (DAB) was detected in plant washings from EGS fields, but worst case scenario estimations suggested the dose was insufficient to cause neurologic disease and DAB was not detected in neural tissues from 6 EGS and 6 control horses. Neither DAB nor the cyanobacterial neurotoxin 2‐amino‐3‐methylaminopropionic acid (BMAA) was detected in neural tissue from 2 EMND horses. *Phormidium* was only apparent in low numbers in plants collected from fields in France where horses had unexplained hepatopathy. While this study did not yield evidence that terrestrial cyanobacterial toxins cause disease in grazing horses, further study is warranted to identify and quantify the toxins produced by cyanobacteria on horse fields, and whether, under appropriate conditions, cyanotoxins contribute to currently unexplained diseases.

## 0029

### GLUCAGON CURVE AND INSULIN‐GLUCAGON MOLAR RATIO IN HEALTHY ADULTS DONKEYS UNDER INTRAVENOUS GLUCOSE CHALLENGE

**F. J. Mendoza^1^, R. Aguilera‐Aguilera^1^, C. A. Gonzalez‐De Cara^1^, R. E. Toribio^2^,J. C. Estepa^1^R. A. Perez‐Ecija^1^**

^1^Department of Animal Medicine and Surgery, University of Cordoba, Campus Rabanales, Ctra. Madrid‐Cadiz km 396, 14104, Cordoba, Spain, ^2^Department of Veterinary Clinical Sciences, The Ohio State University, 601 Vernon Tharp Street, 43210, Columbus, Ohio, USA

Despite limited information on endocrine disorders in donkeys, this species is afflicted by similar disorders as horses. Intravenous glucose tolerance test (IGTT) is widely used in horses in order to diagnose insulin resistance, but this test has not been described in donkeys. In addition, neither the glucagon curve for this glucose challenge or the proxy insulin/glucagon molar ratio (I/G) have been evaluated in this species. The aims of this study were to describe the IGTT, glucagon curve and I/G in healthy donkeys. Seven adults (8.3 ± 1.2 yo) and sixty (6.9 ± 0.7 yo) healthy Andalusian donkeys were used for IGTT and basal I/G calculation respectively. IGTT was carried out in overnight fasted donkeys, via a bolus of 300 mg/kg of 50% glucose intravenously. Plasmatic glucose was determined by spectrophotometry and serum insulin and glucagon concentrations by radioimmunoassay. Donkeys showed a right‐shift of the IGTT curve compared to horses, since glucose returned to baseline later than horses (159.3 ± 12.1 minutes). Insulin concentrations remained significantly higher than baseline longer than horses (175.1 ± 20.2 minutes). Glucagon concentrations decreased rapidly after glucose bolus, but returned to baseline faster than insulin concentrations (122.3 ± 21.2 minutes). During IGTT, I/G ratio increased up to ten‐fold from basal (1.63 ± 0.2), being significantly higher than baseline until 170 minutes. This work is the first characterizing dynamic glucose homeostasis in donkeys via IGTT. Furthermore, clear inter‐species differences have been observed, therefore extrapolating energy metabolism data from horses could lead to misdiagnosis in donkeys.

## 0030

### CLINICAL FINDINGS, TREATMENT AND RESOLUTION OF FLUROXYPYR TOXICITY IN THREE HORSES

**J.I. Michutta^1^, J.M.V. Cavalleri^1^, B. Ohnesorge^1^**

^1^Clinic for Horses, University of Veterinary Medicine Hannover, Foundation, Bünteweg 9, 30559 Hannover, Germany

Three horses were presented after accidental ingestion of Simplex^®^ contaminated stinging nettles to the Clinic for Horses. Upon presentation they were lethargic, moderately tachycardic, with injected mucous membranes and prolonged capillary refill time, bilateral conjunctival hyperaemia, epiphora and a mild to moderate chemosis. Horse 1 also showed a mild ataxia and cardiac arrhythmia. Initial laboratory investigation revealed hyperlactataemia in horses 1 and 2 and an azotaemia in horse 1.

Initially, the stomach was emptied and irrigated with calcium‐gluconate (1%). Thereafter, 5 l of calcium‐gluconate (1%) remained in the stomach. The eyes were flushed with a 10% calcium‐gluconate solution. Intravenous CRI with Ringer's solution was implemented for the first 36 hours and all horses received oral sucralfate (30 mg/kg bwt TID p.o.) over 3 days. Horses 1–3 developed mild hypocalcaemia on hospitalization day 1, oral ulcerations (horses 1 and 2) and a mild skin irritation on the lower lip (horse 1). After resolution of clinical signs and laboratory abnormalities horses were discharged on day 5 (horse 2 and 3) and 6 (horse 1).

The widely used herbicide against broadleaf weeds (e.g. common ragwort), Simplex^®^, contains aminopyralid and fluroxypyr. The fluoride containing fluroxypyr is the more toxic substance, with the kidney as major target organ. We aimed to neutralize the toxic fluoride ions by complexing them with calcium to hardly soluble calcium salts. To the authors’ knowledge this is the first report of a fluroxypyr intoxication in horses. It was successfully treated based on irrigation and supplementation of calcium‐gluconate.

## 0031

### DETECTION OF EQUINE HERPES VIRUS 5 IN DIFFERENT RESPIRATORY SAMPLES OF HORSES

**Leticia Moravszki^1^, Tamas Bakonyi^2^, Peter Miko^1^, Sara Sardi^1^, Zsofia Bohak^1^, Emese Bódai^1^, Orsolya Kutasi^1^**

^1^Equine Department and Clinic, Faculty of Veterinary Sciences, Szent István University, 2225‐H, Üllő Dóra major, Hungary, ^2^Department of Microbiology and Infectious Diseases, Szent István University, Faculty of Veterinary Sciences, 1075 Budapest, István u. 2., Hungary

The aim of our study was to estimate the prevalence of equine herpesvirus 5 (EHV‐5) in Hungarian horse populations, and to evaluate reliability of different respiratory samples in detecting EHV‐5 infection.

Altogether 52 horses were involved in the study, 32 warmbloods from different farms randomly selected and 20 Lipizzaners of a Hungarian National Stud Farm. Six stallions, twenty‐four mares and twenty‐two geldings with the mean age of 8, 17 (sd: 2.09) were sampled. In 36 cases we have collected nasal swabs (NS), peripheral blood leukocyte (PBL) and bronchoalveolar lavage fluid (BALF) samples simultaneously. From 16 horses only NS and PBL samples were collected. Horses were clinically examined and history taken. We have investigated the presence of EHV‐5 in these samples by polymerase chain reaction with the use of virus specific primers.

EHV‐5 nucleic acid was identified in at least one of the investigated samples in twenty horses (38,5%), in 35% of Lippizaners and 40% of warmbloods. All infected horses were positive on NS sample. Both NS and PBL samples were positive in 4 cases and NS and BALF samples showed the presence of EHV‐5 also in 4 horses. Only 2 horses were simultaneously positive on all three samples. All horses positive on BALF had chronic respiratory signs but further diagnostic procedures were not performed.

The results of the study indicate that EHV‐5 is relatively widespread in Hungary. The NS is the most suitable sample for the virus detection. BAL positivity might have a significance in lower airway disorders but further studies are needed.

## 0032

### IN‐VIVO MEASUREMENT OF MUSCLE PROTEIN SYNTHESIS IN THE HORSE

**R.J. Naylor^1^, K. Smith^2^, D. Rankin^2^,V. Blake^1^, P. Atherton^2^, R.J. Piercy^1^**

^1^Comparative Neuromuscular Diseases Laboratory, Royal Veterinary College, London, UnitedKingdom, ^2^University of Nottingham, Derby, United Kingdom

Muscle mass is an important determinant of performance in equine athletes. Furthermore many disease states reduce muscle mass in other species and likely also in the horse. We aimed to evaluate a technique for quantifying MPS *in‐vivo* in the horse using methods that are used in other species, prior to their future application in studies of equine exercise physiology and disease. Five adult male Thoroughbred horses received an infusion of D5‐phenylalanine (D5‐Phe; loading dose 4.5 μmol/kg CRI 3.3 μmol/kg/hr) for seven hours. Skeletal muscle biopsy samples were collect after 1, 3, 5 and 7 hours. After the second biopsy, horses underwent treadmill exercise consisting of 4 mins walk, 4 mins trot and 4 mins canter. The direct incorporation of the stable isotope D5‐Phe into sequential muscle biopsies was quantified using GC‐Pyrolysis‐IRMS. Normally distributed data for fractional synthetic rate was compared between 1–3 hours (baseline), 3–5 hours and 5–7 hours using a repeated measures ANOVA. Basal MPS *in‐vivo* in the horse was 0.049%/hr (SD 0.02), equating to turnover of total muscle mass every 85 days or 4.3 times per year, similar to values previously reported in humans. Treadmill exercise resulted in an increase of mean MPS to 0.080%/hr (SD 0.027) (*P* = 0.14). Measurement of the direct incorporation of D5‐phenylalanine enables measurement of MPS *in‐vivo* in the horse and will be valuable in future studies evaluating MPS in this species.

## 0033

### MICROBIOLOGICAL AND HISTOLOGICAL ANALYSIS OF EQUINE SKIN CORES. IMPACT OF CANNULA SIZE AND INJECTION SITE PREPARATION, FOR INTRAMUSCULAR INJECTION COMPLICATIONS

**T. Puschmann^1^, J. Verspohl^2^, C. Pfarrer^3^, B. Ohnesorge^1^**

^1^Clinic for Horses, University of Veterinary Medicine Hannover, Germany, ^2^Institute for Microbiology, University of Veterinary Medicine Hannover, Germany, ^3^Institute of Anatomy, University of Veterinary Medicine Hannover, Foundation, Bünteweg 9, 30559 Hannover, Germany

The contribution of skin cores, cannula size and preparation protocols to injection site infections after intramuscular injections is discussed, aiming to minimize post injection complications.

This study was designed to characterize histological components of skin cores, and bacterial contamination through skin cores, depending on cannula size (18G, 18G with stylet, 22G) and 4 injection site preparations (*n.prep* = no preparation, *d. prep* = disinfection, *cl.prep* = clipping and disinfection, *as.prep* = antiseptic preparation; *n* = 4 × 10). Skin preparations were obtained antiseptically from euthanized horses. Skin cores were produced by flushing cannulas with saline solution after puncturing the preparations. Cannula contents in culture medium and surface swab samples of all preparations underwent bacterial analysis. Paraffin sections were used for histologic evaluation (*n.prep*).

In 18G (without stylet) cannula contents, bacteria were detected in *n.prep*: 7/10 (10 isolates), *d.prep*: 4/10 (4 isolates), *cl.prep*: 2/10 (2 isolates) and *as.prep*: 2/10 (2 isolates) samples (*P* < 0.05). Comparing cannula sizes (*n.prep*), 18G cannulas with stylet showed the highest detection, rating 9/10 (14 isolates), followed by 18G 7/10 (10 isolates) and 22G 4/10 (6 isolates). In swab samples bacteria were detected in *n.prep*: 10/10 (36 isolates), *d.prep*: 10/10 (16 isolates), *cl.prep*: 10/10 (18 isolates), *as.prep*: 2/10 (2 isolates) (*P* < 0.05). Paraffin sections contained epidermal fractions, with gland and hair elements amongst others.

We conclude that skin cores can transfer bacteria into deeper tissue layers, which may contribute to infective complications post injection. However, the bacterial load can be reduced through appropriate disinfection and usage of smaller cannula sizes.

## 0034

### IDIOPATHIC PERITONITIS IN HORSES: CLINICAL FINDINGS AND LONG TERM SURVIVAL IN 58 CASES

**J. Pringle^1^, Z Lundgren, J Bröjer, P. Haubro‐Andersen**

^1^Department of Clinical Sciences, Swedish University of Agricultural Sciences, 750 07 Uppsala, Sweden

Idiopathic peritonitis in horses, in which no predisposing cause can be identified apart from occasional recovery of *Actinobacillus equuli* in peritoneal fluid, has been reported. Recently, we have observed an apparent increase in admissson of such cases.

Medical records from 2002–2012 were searched for cases of peritonitis. Cases with abdominal surgery or other risk factors such as recent parturition or external/rectal trauma were excluded. Diagnosis was based on peritoneal fluid protein ≥ 30 g/L and/or cells >10 × 109 /L. Individual cases were stratified according to principle diagnosis at admission, including impaction colic or enteritis/colitis, or no gastrointestinal component found, and according to recovery/survival at discharge.

Descriptive statistics were calculated, and the Fishers exact test used for categorical data.

Idiopathic peritonitis was identified in 69 horses but after review of all records for inclusion criteria 58 horses (23 mares, 6 stallions, 29 geldings) aged 11 ± 6.5 years remained. Presenting signs included colic (39/58), fever (45/58), and/or inappetence (35/58). At presentation impaction colic was identified in 20/58 and enteritis in 3/58, whereas no gastrointestinal problems were identifiable in the remaining 35/58. Peritoneal fluid was cytologically positive for bacteria in 11/40 cases, and culture positive in 19/45 cases. Surprisingly, *A equuli* was not commonly isolated.

51/58 cases survived to discharge. However, there was no significant difference in survival dependant on presenting diagnosis or clinical signs.

This work strengthens and extends the growing information on idiopathic peritonitis and supports the suggestion of a good prognosis in cases of idiopathic peritonitis.

## 0035

### A PROSPECTIVE CLINICAL EVALUATION OF THE COMPARATIVE EFFICACY OF THREE TRYPANOCIDES IN THE TREATMENT OF EQUINE TRYPANOSOMIASIS IN THE GAMBIA

**A.G. Raftery^1^, J. Rogers^2^, D.G.M. Sutton ^1^**

^1^Weipers Centre Equine Hospital, School of Veterinary Medicine, College of Medical, Veterinary and Life Sciences, University of Glasgow, ^2^Institute of Infection, Immunity and Inflammation, School of Veterinary Medicine, College of Medical, Veterinary and Life Sciences, University of Glasgow, Bearsden Road, Glasgow, G61 1QH, UnitedKingdom

**Purpose of the study:** To establish relative efficacy of Diminasan^®^, Cymelarsan^®^ and Samorin^®^ in clinical cases of equine trypanosomiasis in a hyperendemic area.

**Methods:** Horses and donkeys were examined (history, clinical examination, blood sample for PCV/TP and PCR) in ten villages in The Gambia. Inclusion criteria were selected to encompass clinical signs correlated to *trypanosoma* sp. infection (two of five: anaemia (PCV<24%), poor body condition (≤1.5), limb or ventral oedema, abortion or pyrexia). Treatments were randomised and follow up was performed at one and two weeks with repeat examination and blood samples. DNA was extracted from a subsection of the blood samples (*n* = 18) in a standard manner (Qiagen^®^ Maxi kit). PCR was performed with primers for *Trypanosoma brucei, Trypanosoma congolense* and *Trypanosoma vivax*. The products were run on gel electrophoresis to identify positive samples.

**Results:** Six hundred and seventy six equines were examined; 254 (145 donkeys and 109 horses) fulfilled the inclusion criteria and received trypanosomiasis treatment. PCV was significantly increased at 1 and 2 weeks post treatment for all groups (*P* < 0.001). The increase was most marked in Diminasan^®^ and Samorin^®^. Preliminary PCR results (*n* = 18) identified 100% (18/18) positive for ≥1 trypanosome before treatment, 33% had mixed infections (6/18). 22% (4/18) remained positive on PCR following treatment.

**Conclusions:** Samorin^®^ and Diminasan^®^ caused the most profound improvement in PCV. Preliminary PCR results confirm suitability of inclusion criteria and support importance of further PCR analysis including pre‐ and post‐treatment qPCR to quantify parasitaemia.

**Practical applications:** The results will provide evidence for selection of an optimal treatment regimen.

## 0036

### NEUROMODULATION USING PERCUTANEOUS ELECTRICAL NERVE STIMULATION; AN EFFECTIVE AND SAFE THERAPY FOR THE MANAGEMENT OF TRIGEMINAL‐MEDIATED HEADSHAKING IN FIVE HORSES

**V.L.H. Roberts^1^, W.H. Tremaine^1^**

^1^School of Veterinary Sciences, University of Bristol, Langford, Somerset, BS40 5DU, United Kingdom

There are no consistently safe and effective methods for the treatment of trigeminal‐mediated headshaking in horses. The trigeminal nerve is hypersensitive, with a lower than normal threshold for activation, which appears to result in neuropathic pain. Percutaneous Electrical Nerve Stimulation (PENS) therapy is a neuromodulatory treatment used in people to manage neuropathic pain. Human patients may experience relief from symptoms following a varied treatment protocol. The protocol assumed by our group is a series of three treatments. Five horses with trigeminal‐mediated headshaking and showing clinical signs were enrolled. All procedures were carried out bilaterally in sedated horses and were tolerated well. The skin over the infraorbital nerves was desensitised with local anaesthetic. A PENS probe was advanced subcutaneously adjacent to the nerve, rostral to the infraorbital foramen under ultrasonographic guidance. The nerve was then stimulated following a protocol of frequency, voltage and duration based on human clinical data. Two horses developed a haematoma at the site on one occasion and two had increased clinical signs for up to three days following first treatment. All horses had a positive response, returning to ridden work. Mean remission time for the first treatment was five days (range three – seven days), for the second treatment four weeks (range zero – eight weeks), for the third 12.5 weeks (range eight – 14 weeks). PENS therapy is a safe well tolerated management option for trigeminal‐mediated headshaking, with encouraging efficacy for amelioration of clinical signs in the short‐medium term.

Ethics – University of Bristol veterinary investigation number 13/022.

Acknowledgements – Coral Winslow‐Llewellyn and Algotec Research and Development for technical expertise and equipment and The Langford Trust for Animal Health and Welfare for funding.

## 0037

### INFLUENCES OF AGE AND SEX ON LEUKOCYTES OF HEALTHY HORSES AND THEIR *EX VIVO*CYTOKINE RELEASE

**C.L. Schnabel^1^, P. Steinig^1^, H.‐J. Schuberth^2^, M. Koy^2^, B. Wagner^3^, B. Wittig^4^, C. Juhls^5^, S. Willenbrock^6^, H. Murua Escobar^6,7^, P. Jaehnig^8^, I. Nolte^6^, C. Pfarrer ^9^, K. Feige^1^, J.‐M. V. Cavalleri^1^**

^1^Foundation, University of Veterinary Medicine Hannover, Clinic for Horses, Bünteweg 9, 30559, Hannover, Germany, ^2^Foundation, Immunology Unit, University of Veterinary Medicine, Bischofsholer Damm 15, 30173, Hannover, Germany, ^3^Department of Population Medicine and Diagnostic Sciences, College of Veterinary Medicine, Cornell University College of Veterinary Medicine, 240 Farrier Rd, Ithaca, New York, 14853, USA, ^4^Foundation Institute Molecular Biology and Bioinformatics, Freie Universitaet Berlin, Berlin, Germany, ^5^Mologen AG, Fabeckstraße 30, 14195 Berlin, Germany, ^6^University of Veterinary Medicine Hannover, Foundation, Small Animal Clinic, Bünteweg 9, 30559 Hannover, Germany, ^7^University of Rostock, Division of Medicine, Clinic III, Hematology, Oncology and Palliative Medicine, 18057 Rostock, Germany, ^8^pj statistics, Niedstr. 16, 12159 Berlin, Germany, ^9^University of Veterinary Medicine, Foundation, Department of Anatomy, Bischofsholer Damm 15, 30173 Hannover, Germany

Leukocytes and their functional capacities are extensively employed as biomarkers in immunological research. Commonly used indicators are leukocyte numbers, composition, response to discrete stimuli, released cytokines and morphometric characteristics.

To employ leukocytes as biomarkers for disease and therapeutic monitoring, physiological variations and influencing factors on the parameters measured have to be considered. The aim of this study was to describe the ranges of selected parameters in healthy horses and to analyze whether age, sex breed, and daytime of sampling influence peripheral blood leukocyte composition, cell morphology and release of cytokines *ex vivo*.

In 24 healthy horses variances of cell size and complexity as measured by flow cytometry were high. Likewise, basal release of selected cytokines by blood mononuclear cells showed a high variability [TNFα (65–16624 pg/mL), IFNγ (4–80 U/mL), IL‐4 (0–5069 pg/mL), IL‐10 (49–1862 pg/mL), IL‐17 (4–1244 U/mL)].

The animals’ age influenced leukocyte composition, cell morphology and cytokine release (TNFα, IL‐4, IL‐10) *ex vivo*. Geldings had smaller monocytes and higher spontaneous production of IL‐10 than mares. The stimulation to spontaneous release ratios of TNFα, IL‐4 and IL‐17 differed in warmblood‐ and thoroughbred‐types. Sampling time influenced leukocyte composition and cell morphology.

In summary, many animal factors – with age being the dominant one – should be considered for studies involving the analysis of equine leukocytes. In addition, high inter‐individual variances argue for individual baseline measurements.

## 0038

### PHARMACOKINETICS OF FIVE COMMERCIALLY AVAILABLE FORMULATIONS OF OMEPRAZOLE

**B.W. Sykes^1^, C. Underwood^1^, C. McGowan^2^, P.C. Mills^1^**

^1^School of Veterinary Science, The University of Queensland, Gatton, 4343, QLD, Australia, ^2^Institute of Ageing and Chronic Disease, Faculty of Health and Life Sciences, University of Liverpool, United Kingdom

Little is published on the pharmacokinetics of different formulations of omeprazole making comparison between products difficult. The objective of this study was to compare the pharmacokinetics of four commercially available formulations of omeprazole to an existing reference formulation. A single dose, cross‐over study was performed using 12 adult Thoroughbred horses. Two generic buffered formulations (OG and AG), one commercial enteric coated formulation (GZ) and one compounded enteric coated formulation (BO) were compared to the reference buffered formulation (GG). Each formulation was administered at a total dose of 2 grams (equivalent to 4 mg/kg for a 500 kg horse). Blood samples were collected at 0, 15, 30, 45, 60, 75, 90, 105, 120, 150, 180, 210, 240, 300, 360 and 540 minutes. Plasma omeprazole concentrations were determined by UPLC‐MS. Non‐compartmental pharmacokinetic analyses were performed using PK Solver and presented as median (IQR). The results of this study, presented below, suggest that modest differences are present between commercially available formulations of omeprazole.

**Table:** 

Formulation	Area‐Under‐the‐Curve (AUC0‐t/0‐inf_obs) (µg/mL*min)	Cmax (µg)	Tmax (min)
OG	45.8 (30.9:60.8)	0.30 (0.20:0.42)	75 (71:83)
AG	41.5 (14.3:59.5)	0.18 (0.10:0.20)	113 (56:180)
GZ	70.4 (43.6:98.1)	0.45 (0.35:0.71)	45 (45:68)
BO	63.7 (38.8:86.0)	0.36 (0.27:0.59)	45 (60:105)
GG	48.3 (45.5:58.0)	0.30 (0.27:0.50)	60 (45:64)

## 0039

### RELATIONSHIP BETWEEN BODY DIMENSION, BODY WEIGHT, AGE, GENDER, BREED AND ECHOCARDIOGRAPHIC DIMENSIONS IN YOUNG ENDURANCE HORSES

**D.S. Trachsel^1,2^, A. Giraudet^2^, G.Hervé^2^, D. Maso^2^, D.D. Hauri^3^, E. Barrey^4^, C. Robert^2,4^**

^1^CIRALE‐Hippolia, Médecine Sportive, RD 674, 14430 Goustranville, France, ^2^Ecole Nationale Vétérinaire d'Alfort, 7 avenue du Général de Gaulle, 94704 Maisons‐Alfort, France, ^3^Office Fédéral de la Statistique, Espace de l'Europe 10, 2010 Neuchâtel, Switzerland, ^4^INRA, GABI‐UMR1313, Jouy‐en‐Josas, France

Heart dimensions are related to body weight (BWT), body size, and growth. Training, especially endurance training, also influences the ventricular size. Further, breed is relevant to determine normal heart dimensions in certain species. However, few data on the evolution of heart dimensions with growth and training in Arabian and Arabian related horses are available.

Therefore, the study aimed at describing the effect of body dimensions (body length, thoracic perimeter (TP), withers height (WH)), BWT, age, gender and breed (pure breed, Arabian cross, anglo‐arabian, others) on echocardiographic measurements in competition fit endurance horses aged 4–6 years. Standardised echocardiographic images of 302 left ventricles (LV) and 256 left atria (LA) and great vessels were obtained at rest by two experimented examiners. The relationship between echocardiographic measurements and mentioned predictors was assessed by multiple linear regression models with selection of relevant predictors based on the adjusted R^2^ and AKAIKEI's information criteria.

BWT and WH showed a positive influence on most LV 2D and area dimensions. Age influenced LV area, volumes and functional indices, gender influenced LV area, volumes and mass, whereas breed influenced LV internal diameter and mass. All LA and great vessels dimensions increased with increasing body dimensions, especially TP. Age had an influence on all 2D dimensions, whereas BWT, gender and breed affected only some of them.

In conclusion, BWT and body dimensions as well as age, gender and breed are important for assessing cardiac dimensions and should be considered when establishing normal values for Arabian type horses.

## 0040

### SUCCESSFULL TREATMENT OF DERMATOGRAPHISM WITH CETIRIZINE IN A HORSE.

**A.J. Van den Brom‐Spierenburg^1^, M.J.P. Theelen^1^, M.M. Sloet van Oldruitenborgh‐Oosterbaan^1^**

^1^Department of Equine Sciences, Faculty of Veterinary Medicine, Utrecht University, Yalelaan 112, 3584 CM Utrecht, the Netherlands

Dermatographism is a common condition in humans. Local pressure applied to the skin triggers mast cells to release histamine, causing pruritic urticaria on the pressure‐spots. In horses only one case of dermatographism was reported in 1989 describing an 8‐year‐old Thoroughbred gelding.

This case report is about a 4‐year‐old Quarter horse gelding that was presented with the complaint of pressure induced urticaria. When pressure was applied to the skin non pruritic urticaria‐like swelling was evident after approximately ten minutes and this lasted for approximately two to three hours. It was indeed possible to ‘write’ on the horse. On clipped skin the condition was less evident.

Based on extrapolation of human literature mast cell involvement was suspected, and the horse was treated with cyproheptadine (0.3 mg/kg q12 h). This showed to have some effect by reducing clinical signs. However, the horse developed side‐effects consisting of ulceration of the gingiva and significant dullness. Therefore therapy was changed to cetirizine (0.2 mg/kg q12 h). With this treatment no side‐effects were noticed and dermatographism was no longer present. After a few months the treatment was discontinued and clinical signs did not reoccur. Presently it is still too early to determine a possible seasonal influence.

This case report shows that dermatographism in horses occurs and can be treated successfully with cetirizine.

## 0041

### ULTRASOUND‐GUIDED PERCUTANEOUS TRANSCATHETER DELIVERY OF AN OCCLUSION DEVICE IN TWO HORSES

**G. vanLoon^1^, D. De Clercq^1^, A. Decloedt ^1^, S. Ven^1^, N. Van Der Vekens^1^, M. Jordana^2^, Y. Taeymans^3^ , D. De Wolf^3^**

^1^Departments of Large Animal Internal Medicine, Ghent University, Belgium, ^2^Surgery and Anaesthesiology, Faculty of Veterinary Medicine, Ghent University, Belgium, ^3^Department of Pediatrics and medical genetics, Faculty of Medicine and Health Sciences, Ghent University, Belgium

In human and small animal medicine, intracardiac or vascular shunt closure is often performed with an occluder, delivered via a catheter. Fluoroscopy is mandatory to guide the procedure. In adult horses, fluoroscopy cannot be used for cardiac imaging due to body size. The aim of the study was to perform an ultrasound‐guided delivery of a cardiovascular occlusion device. An Occlutech Figulla ASD^®^ device was delivered via a common carotid artery approach under general anaesthesia, guided by parasternal ultrasound. For catheter guidance during aortopulmonary fistula and an aortocardiac fistula closure, ultrasound was performed from a left and right parasternal view, respectively. Guide‐wire, delivery catheter (14F, Mullins Transseptal Check‐Flo Introducer^®^) and occluder appeared hyperechogenic on ultrasound and were successfully manoeuvred through the shunt, after which the occluder was deployed into the shunt. The occluder in the aortopulmonary fistula embolised as the fistula was much larger (65 mm) than the largest available occluder (post mortem result). The device in the 27 mm diameter aortocardiac fistula was successfully deployed and immediately improved the hemodynamics and clinical signs of the horse. Recovery was uneventful but dislodgement occurred after one week. In conclusion, ultrasound‐guided transcatheter delivery of an occlusion device is feasible in adult horses. Occluder dislodgement occurred in both horses probably due to a poor matching of the device to the shunt and the relatively soft material probably not perfectly suited for the cardiovascular hemodynamics in the horse. We concluded that ultrasound‐guided transcatheter shunt occlusion is feasible but probably more appropriate devices are needed.

